# Sex Doll Ownership: An Agenda for Research

**DOI:** 10.1007/s11920-020-01177-w

**Published:** 2020-08-15

**Authors:** Craig A. Harper, Rebecca Lievesley

**Affiliations:** Department of Psychology, Nottingham Trent University, 50 Shakespeare Street, Nottingham, NG1 4FQ UK

**Keywords:** Sex dolls, Sexuality, Sex robots, Sex offending, Sexual abuse prevention

## Abstract

**Purpose of Review:**

The topic of sex doll ownership is becoming an increasingly discussed issue from both a social and legal perspective. This review aims to examine the veracity of the existing psychological, sexological, and legal literature in relation to doll ownership.

**Recent Findings:**

Strong views exist across the spectrum of potential socio-legal positions on sex doll ownership. However, there is an almost total lack of empirical analyses of the psychological characteristics or behavioral implications of doll ownership. As such, existing arguments appear to represent the philosophical positions of those scholars expressing them, rather than being rooted in any objective evidence base.

**Summary:**

Despite an absence of empirical data on the characteristics and subsequent effects of doll ownership, discussions about the ethical and legal status of doll ownership continue. This highlights a real and urgent need for a coherent research agenda to be advanced in this area of work.

## Introduction

The sale of realistic human-like dolls designed for sexual use is a multi-million dollar global industry [1]. Newer doll models are fully customizable, and markets are opening up that allow for dolls to be modeled on real people, including adult film stars [2]. Some models include artificial intelligence, and the ability to feign communication with their owners (referred to as sex robots). The emergent scale of sex doll and robot ownership has led to increasing amounts of academic, social, and legal attention being directed towards this topic. The latter of these domains—the law—is increasingly important with growing levels of attention being directed towards child-like sex dolls, after a number of people have been convicted for the importation of such objects [3•, 4–6]. In spite of this growing attention, there exists no review of the empirical academic evidence about the motivations and effects of doll ownership. While a small number of papers and governmental reports have been published that purport to do this, these are typically short in length, conclude there is no empirical evidence about doll ownership, and advocate for restriction of doll sales and availability until evidence does exist [3•, 7•]. In this paper, our aim is to review the key arguments in relation to the motivations and effects of doll ownership, but go further than other papers by offering a critical evaluation of these arguments. In doing so, we build towards some research questions and hypotheses that we believe should form the basis of future research into sex doll and robot ownership.

## Who Are (Potential) Sex Doll Owners?

As it currently stands, the literature around sex doll and robot ownership appears to suggest that these objects have an overwhelmingly sexual function [8–11]. While this may be true to some degree, survey data suggest that purely viewing dolls and robots of this type through a sexual lens may limit our understanding of this phenomenon. For example, while up to 70% of sex doll owners cite sexual gratification as the primary function of their doll, others discuss how their dolls act as a form of friendship and companionship [1, 2]. In addition, even among those who did suggest that sex was their doll’s primary purpose, this was accompanied by other functions in more than 80% of cases [1, 12]. In perhaps the most thorough analysis of doll owner motivations, Su and colleagues analyzed the forum posts originating from more than 5500 threads on the world’s largest forum for sex doll owners [13••]. This led to a corpus of almost 80,000 posts over a timeframe of 14 years, from 2001 to 2015. Coding these posts, a range of potential motivations emerged. In addition to purely sexual motivations (i.e., the availability of a “willing” and available sexual partner whenever sexual gratification is desired), owners used the forum to discuss using their dolls’ artistic functions (e.g., photography), as well as how they created meaningful intimate relationships with them. It is in relation to this latter function of dolls where they become anthropomorphized. At the beginning of their journey, doll owners talk about the adoption of their new companion, with them creating identities for them through the application of realistic tattoo transfers and makeup. Dolls are spoken about in terms that empower them and give them a voice. According to Su and colleagues, this provides owners with the illusion of intimacy, which forms the basis of a close bond between them and their dolls.

This range of relationship types between humans on the one hand, and dolls and robots on the other, is reflected in media representations. That is, human owners are typically depicted as emotionally disadvantaged and their robot partners take the form of a source of sexual gratification and emotional intimacy [14]. While these themes are present in fictional depictions of doll ownership, they are also reflected in studies of non-owners who are asked about their intentions to obtain a sex doll or robot, or the appeal of owning such objects. For example, men scoring high on indices of otakuism (operationalized as being shy, reserved, and having a preference for indoor activities over outdoor pursuits) show an increased behavioral intention to own a sex robot [15]. Other work has found that a fear of rejection is associated with enhanced ratings of robot attractiveness [16], suggesting that interpersonal deficits may be predictive of a proclivity to engage with sex dolls and robots.

There may be some individuals who become sex doll or robot owners for medical or psychotherapeutic reasons. According to data published by Eichenberg and colleagues, around two-thirds of clinical staff working in the area of sex therapy may foresee some utility of incorporating dolls into their practice [17]. Predictions about the efficacy of dolls in these settings differ, however, with therapists suggesting that dolls might be more appropriate for use with people who have sexual anxiety and erectile dysfunction than for those trying to increase satisfaction with their living partners, or who are struggling with problematic sexual arousal. Linked to this, older people in care homes and those with physical and intellectual disabilities who have unmet sexual needs may also benefit from the incorporation of sex dolls and robots into their care plans [18]. Others have suggested a role for sex dolls in initiatives designed for the prevention of sexually transmitted infections [19], highlighting the broad range of uses for these models in healthcare and education settings.

## The Alleged Implications and Effects of Doll Ownership

An overwhelming proportion of the existing published scholarship on sex doll and robot ownership addresses, or at least seeks to address, the ethics of owning and engaging with sex dolls and robots. For example, scholars in bioethics, sociology, robotics, and legal studies have all cited concerns that sex dolls and robots encourage the sexual objectification of women and exacerbate traditional standards of beauty and perceptions of attractiveness [9, 10, 15, 20–24], with the ultimate effect of this being a loss of human intimacy and connection when it comes to sexual interactions [24–26]. At its extreme, this collection of work suggests that doll and robot ownership has the effect of promoting sexual violence and child sexual abuse, which in much of this work is erroneously synonymized as pedophilia [4, 6, 27, 28]. While many of these claims make sense at face value, their empirical bases are open to challenge.

Emerging theoretical models of objectification suggest that this process involves the viewing of a particular entity as a means to an end, meaning that a defining feature of objectification is instrumentality [22, 29–32]. In delineating the nature of objectification, Orehek and Weaverling suggest that there is nothing about objectification (as a psychological process) that is inherently moral or immoral. In fact, objectification may even be an inevitable mental process, as “to suggest that people should not be objectified is to say that they should not be evaluated” [29]. In this regard, the extent to which objectification becomes dependent on some other factor(s), such as the underpinning values of the person who is labeling something as objectifying, or the behavioral implications of the objectifying cognitions or acts. That is, if objectification (specifically of a doll in the current context, or of women more generally as a result of doll ownership) has no behavioral effects (e.g., an increased proclivity for sexual aggression), then neither doll ownership nor the resultant objectification can be considered to be immoral.

There is a large literature on how the sexual objectification of women predicts more permissive attitudes towards sexual aggression [33–35]. However, it is important to stress that there is currently no empirical evidence that doll ownership translates into elevated levels of sexual objectification, either of dolls as objects themselves or of real women. Indeed, applying data from media effects research in related areas would suggest that concerns about the transferability of violence from various forms of mass media to personal behavior might be misplaced. Recent analyses of the effects of playing violent video games [36, 37] and, more relevant to the sex dolls and robots, the viewing of pornography, suggest that engaging with these media has little effect on behaviors in the real world among those with no pre-existing aggressive propensities [38, 39]. More than this, the evidence for the effects of pornography appear to show a potentially cathartic effect, where societies that have higher rates of pornography use demonstrate lower rates of sexual violence [39, 40].

Linked to the topic of objectification is that of gendered stereotypes about what constitutes beauty and sexual attractiveness. According to a thesis by Krizia Puig, female-like sex dolls represent a form of synthetic hyper femininity that reinforces “whiteness, thinness, being cis-gender and being heterosexual as what is considered desirable and beautiful” [41]. While this argument self-evidently stems from the epistemological tradition of intersectional queer theory, it does highlight the view that the physical characteristics reflect particular features that are designed to attract male buyers. One suggestion to address this issue of a reinforcement of a single beauty standard has been to abandon the “unsophisticated” porn star design of sex dolls and robots, and to instead create “robots that are more realistic in their representations (both physical and behavioral) of women, that represent men, and that perhaps challenge the gender binary” [10].

While several authors have suggested that these features reinforce socially constructed notions of beauty [9, 10, 25, 41], insights from evolutionary psychology tell a different story. Gad Saad has pioneered the field of evolutionary consumption, the field that applies evolutionary psychological theory to the study of consumer behavior. He has presented a nomological network of cumulative evidence examining the argument for an evolved male sexual preference for women with an hourglass body type [42]. These networks consider a range of evidence from disciplines that appear to converge on a consistent conclusion. In the case of an hourglass figure preference for women among men, Saad presented evidence that the hourglass figure confers biological cues suggestive of youth and fertility [43, 44], resulting in a cross-cultural sexual preference among men using a range of psychophysiological outcomes [45–47]. In the sexual services domain, an hourglass figure (operationalized as a waist-to-hip ratio of approximately 0.70) is consistently reported by female sex workers in their online service advertisements [48], with a figure more closely matching this ratio being associated with a higher fee being charged by female escorts [49]. Specifically related to sex dolls, adult-sized models have been reported to have an average waist-to-hip ratio of 0.68 [50]. In light of these data, the argument that sex dolls reinforce socially constructed beauty ideals is challenged by the competing view that dolls simply reflect an evolved male preference for a particular body type, and are thus driven by market demand. In short, customer preference drives model design, rather than vice versa. Nonetheless, longitudinal studies investigating the effects of exposure to sex dolls on indices of objectification and body type preferences would be a useful starting point for exploring the relative validity of these competing hypotheses.

As already intimated, there is no research that directly examines a causal link between sex doll ownership and a proclivity to engage in sexual aggression. Instead, the existing literature hints towards these links from philosophical, ideological, and ethical positions [9, 20–22, 28, 51]. Absent this causal evidence, some philosophical arguments have gone so far as to consider whether sex with robots should be considered rape in-and-of itself [4, 27]. For instance, a concept piece led by Elen Carvalho Nasciemento stated that “sex robots exploit the female figure - eventually male, and perhaps even children - for unilateral physical pleasure. The buyer of the object, a humanized sex toy, possesses these bodies to do what they want, with no need of consent” [20]. Sinziana Gutiu went further, suggesting that the programmable nature of sex robots allows for their owners to in essence practice the act of rape:

… the sex robot looks and feels like a real woman who is programmed into submission and which functions as a tool for sexual purposes. The sex robot is an ever-consenting sexual partner and the user has full control of the robot and the sexual interaction. By circumventing any need for consent, sex robots eliminate the need for communication, mutual respect, and compromise in the sexual relationship. The use of sex robots results in the dehumanization of sex and intimacy by allowing users to physically act out rape fantasies and confirm rape myths. [51]

Extending this line of argument still further, John Danaher offered a tentative argument in favor of criminalizing sex robots as tools for “robotic rape and robotic child abuse” [4]. He does this by considering the potential social effects of sex robots (see above), before logically pulling together two premises: that morally wrongful conduct can be criminalized in law, even when no objective harm to others is caused (the *moralistic premise*), and that robotic rape and child abuse represent morally wrongful conduct (the *wrongness premise*). However, Danaher offers no coherent explanation as to why engaging in sexual activity with a humanoid robot should constitute “rape” or “child abuse” in its own right, or in a specific sense.

Eskens addressed this issue more directly, concluding that sex with robots does not constitute “rape” in the strictest legal sense, as the normative use of this term involves a consideration of consent. She concluded that robots lack moral value (by virtue of their artificiality and lack of cognitive capacity (see also [52, 53]), and thus cannot give (or withhold) consent, and by extension cannot be victims. In spite of setting out these arguments, Eskens tempered her argument by stating that “although it might not be impermissible to have sex with robots for the reason that it is non-consensual, it might be impermissible for other reasons,” invoking those ideas of objectification, desensitization, and the promotion of offense-supportive cognitions, as described above.

## Child-Like Sex Dolls, Pedophilia, and Child Sexual Abuse

The philosopher John Danaher examined the issue of child sex doll and robot criminalization using the perspective of legal moralism [5]. In essence, this position posits that the immorality of a particular behavior or object should be able to serve as a basis for its restriction or criminalization, irrespective of the objective harm (or lack thereof) that it causes other people. Danaher draws upon the work of Strikwerda to argue that engaging in sexual activity with a child-like sex doll or robot encourages and normalizes a non-virtuous form of sexual activity, owing to its ostensibly non-consensual and power-imbalanced nature [6]. In other work, Danaher has gone so far as to argue that owning child sex dolls and robots indicates a lack of moral virtue in its own right [4], suggesting that one potential consequence of ownership could be inclusion in registration processes currently reserved for individuals with sexual convictions. Danaher is not alone in theorizing about child-like dolls and robots from such a moralistic position.

In a further attempt to justify the criminalization of the creation and distribution of child-like sex dolls and robots, Chatterjee also acknowledged the lack of objective personal harm caused by these materials. However, she invoked reasoning related to “cultural harm” to argue that “permitting a trade in even abstract child sex dolls and robots could be seen as sanctioning and facilitating a public atmosphere that encourages the portrayal of children as sexual objects, and the acceptance and normalization of child abuse” [54]. While this argument makes logical sense, it is telling (and reflective of the rest of the philosophical literature on child sex dolls and robots) that it is presented in isolation and without references to an opposing view related to child sexual abuse prevention. Some professionals working within forensic health services have tentatively suggested that child-like dolls and robots could play a role in the prevention of child sexual abuse (for discussions, see [3•, 5, 54]). This argument suggests that dolls and robots offer a sexual outlet without a victim that could satiate the sexual fantasies of some individuals with sexual interests in children. While this possibility is mentioned in some of the philosophical papers cited above, it is quickly dismissed with doubt, possibly due to an inherent misunderstanding of the nature of such sexual interests within the philosophical community (see [4, 5, 54, 55]) without any serious empirical consideration of its merits taking place.

The Australian Institute of Criminology (which operates as a branch of the Australian Government) commissioned a report, authored by Rick Brown and Jane Shelling, into the potential behavioral and legal implications of child sex doll use [3•]. In it, the authors purported to review the available evidence on child sex doll use and proposed recommendations on the basis of this review. Specifically, the implications under investigation included the following:

1. the promotion of the sexualization of children;
2. the extent to which child-like sex doll use is an indicator of an escalation from using child sexual exploitation material (CSEM);
3. the normalization of child sexual abuse (i.e., the causal link between child-like sex doll use and contact sexual offending against children); and
4. the risk that child-like sex dolls can be used as a tool for the sexual grooming of children.

Throughout the report, Brown and Shelling identify no empirical evidence for any of these hypotheses about the effects of child-like doll ownership. This was explicitly stated in sections about the harmfulness of doll ownership (i.e., promoting of sexual offending), while the legal moralism perspective of philosophical authors [4–6, 28] was invoked once again when discussing the implications of dolls on objectification and desensitization to themes of child sexual abuse. In a comparatively short section of their report, they also discussed the possibility of child-sex sex dolls playing a role in the prevention of sexual abuse, again highlighting the dearth of empirical data that directly tests this hypothesis. It is remarkable, then, given this explicit lack of empirical data, that Brown and Shelling conclude:

It is reasonable to assume that interaction with child sex dolls could increase the likelihood of child sexual abuse by desensitizing the doll user to the physical, emotional and psychological harm caused by child sexual abuse and normalizing the behavior in the mind of the abuser. At the same time, there is no evidence of therapeutic benefit from child sex doll use. [3•]

Most surprising here is not the assertion that it is perhaps reasonable to assume a degree of sexual risk among some people who own child-like sex dolls (see also [5, 6, 54, 55]). Rather, it is more concerning, and perhaps illuminating of this area of scholarship, that this conclusion is reached alongside one pertaining to their lack of therapeutic benefit in light of the total lack of evidence in either direction. This is perhaps reflective of the nature of the authors’ theoretical understanding about the nature of sexual offending. That is, they cite feminist and sociological conceptualizations of sexual offending by arguing that this behavior is driven by primarily by a desire to exert dominance, power, and control, and not the achievement of sexual satisfaction [26, 56, 57]. While a detailed exploration of this argument is not specifically within the remit of this review, we direct readers to each of the major multifactorial models of sexual offending used within forensic psychological research and practice; all of which contain sexual interest or arousal as a key causal component [58–63].

This approach was also taken in a review article published by Chantal Cox-George and Susan Bewley, who sought to provide a succinct summary of the arguments for and against the sex robot industry and to assess the potential health implications that may affect both patient behaviors and clinicians’ professional activity [7•]. Consistent with Brown and Shelling’s report in Australia [3•], Cox-George and Bewley discussed the potential risks of using sex dolls and robots in clinical settings, despite their literature search yielding no empirical studies for them to review. The reasons for these authors taking such a defensive position included police interest in dolls, potential clinical prosecutions for providing illicit materials, and the broader possibility of negative public responses to the use of dolls and robots in clinical settings. This led Cox-George and Bewley to advise against incorporating dolls and robots into clinical practice, as this alleviates the danger of professionals facing repercussions because of their use.

While the widespread adoption of child-like dolls in forensic and clinical settings in the absence of evidence should not be promoted, these reviews do suggest a skewed reading of the literature (for a commentary, see [64]). It is incumbent on scientists, clinicians, and policy makers to maintain an open mind on the potential for both benefits and risks until evidence is available. We now turn our attention to what the beginnings of a comprehensive research agenda into sex dolls and robots might look like.

## Towards an Agenda for Research

In light of the almost complete absence of empirical research into the effects of sex dolls and robots on owner attitudes and behaviors, we close our review with a call for research into these crucial outcomes. Specifically, research is needed in several domains, including the following:

1. The different motivations of sex doll and robot owners
2. The effects of sex dolls and robots on sexual cognition and behavior (e.g., sexual fantasy and aggression)
3. Social attitudes and responses to sex dolls and robots

Considering analyses of qualitative accounts provided by sex doll and robot owners in existing work [2, 13••], deeper and more specific analyses of the motivations of owners for obtaining dolls and robots should be conducted. While the existing work provides a useful starting point, these papers are limited by the nature of the data collected. That is, survey-based and forum data are limited in relation to the level of explanatory depth that can be obtained. Future studies may look to conduct interviews with doll and robot owners. This approach allows for a conversation to be had between the researcher and the subject, providing the opportunity for deep phenomenological accounts about the nuanced functions of dolls and robots to be uncovered. This level of analysis may also uncover new insights about the types of quantifiable variables that may distinguish doll and robot owners from those who do not own such objects.

The overwhelming conclusion that emerges from the existing literature on sex dolls and robots is that there is an almost total lack of empirical evidence about the psychological and behavioral effects of owning these objects. A propensity to engage in increased levels of the sexual objectification of women, the strengthening of rigid standards of sexual attraction, and an increased risk of sexual offending are all cited as potential implications of doll ownership [3•, 7•, 20, 21, 23, 28, 51]. However, while these arguments make logical sense, their empirical validity remains untested. Research is urgently needed to test these assertions. Initially these studies may take the form of cross-sectional designs exploring whether levels of sexual objectification or offending proclivity differ between groups of sex doll owners and non-owner controls. If differences are discovered, then longitudinal studies investigating whether doll and robot ownership is causally linked to elevated risk levels would be required. That is, non-owners who are considering doll ownership might be followed-up in order to explore how (or perhaps whether) they change with respect to indices of potential sexual aggression. Irrespective of the results of cross-sectional surveys of doll and robot owners, multifactorial theories of sexual offending could play an important role in determining the conditions under which such ownership is or is not “safe.” Seto’s motivation–facilitation model, for example, asserts that a sexual interest may lead to a psychological disposition towards engaging in an offending behavior, but that the actual enactment of such behaviors are facilitated (or hindered) by the presence (or absence) of facilitating factors, such as antisocial personality traits or dependency on drugs or alcohol [63]. In applying this to the doll and robot context, sexual offending proclivity may be predicted via an interaction between sex doll ownership (a potentially motivating sexual interest) and the presence (or absence) of offense-facilitating (or offense-reducing) personality and lifestyle factors.

In addition to the potential risks associated with doll and robot ownership, research should also investigate the various positive effects that doll ownership may yield. Controlled access to dolls in sex and relationship counseling settings may be a useful starting point for this, so as to explore the effectiveness of dolls in alleviating sexual dysfunction. More controversially, trials of child-like sex dolls may be considered in settings geared towards the prevention of sexual abuse among individuals who are sexually attracted to children. Case study evidence of this practice should be considered and evaluated prior to larger trials of the efficacy of dolls as a cathartic sexual outlet for minor-attracted persons. However, such trials are the only meaningful way to address the controversies and unanswered questions currently posed in the literature.

As highlighted previously, sex dolls and robots have a potential role to play in medical and psychotherapeutic settings for alleviating feelings of social and emotional isolation, or in the treatment of sexual dysfunction [11, 17]. However, at present it is acknowledged that social responses to doll and robot ownership may act as a blockage to such usage, even if there was evidence of its effectiveness [7•]. There is minimal evidence about the nature of social attitudes regarding sex doll and robot ownership. In the data that are available, men are typically more accepting of doll ownership and use in clinical settings [15, 17], as are younger participants [17]. Higher levels of religiosity have also been associated with more negative attitudes [65], which may be reflective of the view that such objects are not a creation of God. However, there currently exists no standardized measure of attitudes towards sex dolls and robots, as well as their owners. The development of such a measure, potentially using inspiration from scales designed to examine stigmatization of other sexual minority groups, will enable the systematic study of such responses, and help to identify the types of factors that alter social perceptions of this topic. Such factors may include the apparent age of the doll (adult-like vs. child-like), the function it is designed to fulfill (e.g., sexual gratification vs. the treatment of mental distress), or the personality traits of doll and robot owners (e.g., levels of psychopathy vs. empathy).

## Conclusions

Our aim in this review has been to chart the development of arguments emerging within the existing literature on the motivations and effects of sex doll and sex robot ownership. We further sought to critique these ideas in light of the dearth of empirical research into this topic, using insights from social, cognitive, and evolutionary psychology, as well as contemporary theorizing about the etiology of sexual offending. In doing so, we have highlighted how the conclusions reached by scholars currently writing about this topic are borne out of ideological and risk-averse starting positions, with this sometimes being explicitly acknowledged by these authors. We have presented some initial ideas for a research program into the motivations of sex doll and robot ownership, the behavioral implications of such ownership practices, and social responses to individuals who may wish to own a sex doll or robot. It is our hope that social scientists and legal scholars will use these ideas to engage in more empirical research into this increasingly contentious social issue.
